# Rational Use of Antibiotics in the Treatment of Functional Bowel Disorders

**DOI:** 10.3390/ph3082380

**Published:** 2010-07-26

**Authors:** Michele Di Stefano, Roberta Fasulo, Gino Roberto Corazza

**Affiliations:** Department of Medicine, University of Pavia, Foundation IRCCS S.Matteo Hospital, P.le C. Golgi 2, Pavia, Italy

**Keywords:** small intestine bacterial overgrowth, functional bowel disorders, irritable bowel syndrome, rifaximin, gas-related symptoms

## Abstract

Functional gastrointestinal symptoms such us bloating, fullness, flatulence, diarrhea, and constipation due to irritable bowel syndrome (IBS) were recently attributed to small bowel bacterial overgrowth, a condition depending on the presence of an increased number of bacteria in the small bowel. However, the methodology used to describe this association may be harshly criticized, since it has already been shown to be quite inaccurate. As a result an inappropriate use of antibiotics was consequently generated. In fact, antibiotics could be effective in the treatment of functional complaints, but only in a limited subgroup of patients, characterized by an increase of fermentation at colonic level. In this review, we have examined the papers suggesting a pathophysiological link between IBS and small bowel bacterial overgrowth, underlining its inappropriateness, and put forth our personal view on the rationale for antibiotic use in IBS.

## 1. Introduction

It has been estimated that functional bowel disorders are characterized by a high prevalence in the general population [1] and they represent a very frequent cause for gastroenterological outpatient consultation [2]. Apart from pain and the modification of bowel habit, patients with irritable bowel syndrome (IBS) present a wide range of symptoms and co-morbidities, recently extensively reviewed as regards both prevalence and pathophysiology [3,4,5]. As far as symptoms are concerned, the most frequent are abdominal pain/discomfort, abdominal bloating, straining, incomplete evacuation and urgency [5]. In particular, bloating may represent a very bothersome symptom and may be present in up to 80-90% of patients [6,7,8,9,10]. Another annoying symptom is flatulence, also very frequent in patients with IBS [11] and not always associated with bloating. 

## 2. Intestinal Gas and Symptoms

Bloating and flatulence are commonly considered as caused by an impaired intraluminal gas production and handling, but their pathophysiology is certainly multifactorial. The reduction of excessive colonic gas production improves bloating and flatulence severity [12,13], but bloating is frequently present in conditions characterized by an obstruction of luminal flow and the restoration of normal transit is followed by symptom relief [14]. Moreover, abnormal wall muscular activity may also cause bloating, as a failure of abdominal muscle contraction together with a paradoxical relaxation of the internal oblique muscle occurring during colonic infusion of gas [15]. However, the putative pathophysiological role of intraluminal gas is suggested by the improvement of both bloating and flatulence severity after pharmacological stimulation of intestinal peristaltic activity, inducing an increased elimination of intraluminal gas [16]. It is, therefore, conceivable that at least in a subgroup of functional patients intraluminal gas plays a role in the onset of bloating and flatulence. 

Levitt and coworkers [17] analyzed the relationship between abdominal bloating and intestinal gas by supplementing healthy volunteers’ diet with fermentable (lactulose) or non-fermentable (methylcellulose) carbohydrates or placebo for a 1-week period. As far as passage of flatus is concerned, lactulose supplementation increased its frequency, while no difference was seen after administration of methylcellulose or placebo. On the contrary, both lactulose and methylcellulose caused an increase in abdominal bloating scores. This finding could be explained on the basis of the increased fecal bulk by methylcellulose and on the enhanced gas production by lactulose. Both mechanisms are able to induce a stimulation of bowel wall mechanoceptors due to increased intraluminal pressure and subsequent enhancement of wall tension in the involved GI tract [18]. Consequently, flatulence certainly represents a gas-related symptom, but abdominal bloating is not always related to an increased gas production. 

Accordingly, these observations are extremely important for the selection of patients who might obtain a positive effect on symptom severity after treatment aimed at the modification of intestinal gas production. On these grounds, the use of antibiotics in the treatment of functional bowel disorders has received growing attention. 

## 3. Clinical Overlap between Small Intestine Bacterial Overgrowth and IBS

Small Intestine Bacterial Overgrowth (SIBO) is a condition defined by the presence of pathological amounts or types of bacteria at the small bowel level, responsible for a malabsorption syndrome, clinically evident through a spectrum of symptoms such as diarrhea, flatulence, abdominal pain, bloating, and, in more severe cases, anaemia, weight and bone loss [19,20]. Bacterial overgrowth is associated with the presence of several predisposing conditions: conditions causing stasis, gastric resections with reduced efficacy of “gastric acid filter” as well as intestinal resection with altered efficacy of the ileo-cecal valve [19,20]. Although characterized by very different pathophysiology, all these conditions are responsible for allowing bacteria to pass into or develop in the small bowel. A predisposing condition is crucial for SIBO to occur. Indeed, its prevalence in patients with one of these conditions is extremely high and larger than that seen in patients without [21]. 

SIBO presents a wide clinical overlap with IBS: consequently, a pathophysiological link between these two conditions was suggested: in particular, it has been wrongly suggested that IBS is caused by bacterial overgrowth in the small intestine.

## 4. The Inaccuracy of the Lactulose Breath Test to Diagnose SIBO

The association between IBS and SIBO is based on a largely incorrect interpretation of the lactulose breath test [22]. The use of the lactulose breath test to diagnose SIBO was first described by Rodhes *et al*. [23], but its inaccuracy became rapidly evident and the same authors published a revision of the diagnostic criteria a few years later [24]. Nevertheless, the accuracy remained inadequate and the association of the lactulose breath test and scintigraphic methods also proved to be inaccurate [25]. Our experience too is in line with these results [21,26].

**Figure 1:**
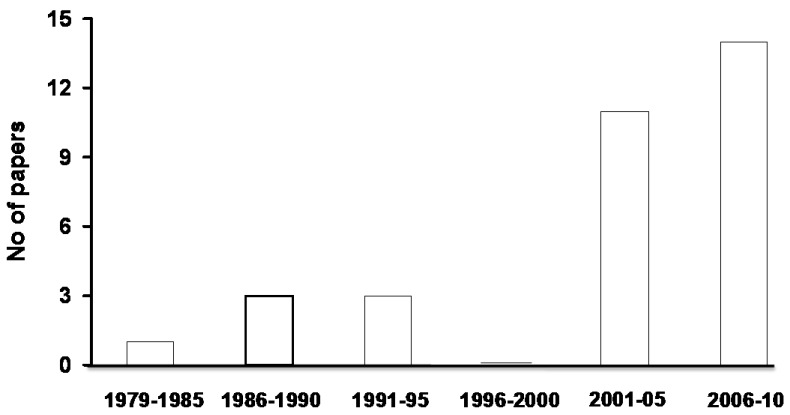
Number of papers in peer-review literature adopting lactulose breath test to diagnose SIBO, together with the year of publication.

Although these demonstrations were obtained some 30 years ago, scientific articles reporting the use of the lactulose breath test to diagnose SIBO are still being published in 2010. Figure 1 reports the number of papers using such a wrong method, subdivided according to the year of publication.

The first study suggesting a role for contaminating flora of the small intestine in the pathophysiology of IBS reported, according to this wrong interpretation of results, a prevalence of 78% of SIBO in IBS. Moreover, when antibiotic treatment induced an eradication of bacterial overgrowth, an improvement of symptoms was evident [22]. In this study, only patients with diarrhea were enrolled, thus suggesting an important interference due to an accelerated transit time, causing the wrong interpretation of breath test results. Moreover, the improvement of symptoms after antibiotic treatment could have been induced by the effect of antibiotics on colonic flora, rather than small bowel contaminating flora which, in our opinion, was not present in the cohort of patients. The results of the other two studies showing a significant symptomatic improvement after metronidazole [27] and neomycin [28] administration could be interpreted in the same way. 

The interfering effect of an accelerated transit time is imputable also when the glucose breath test is adopted. In a recent paper, [29] only patients with IBS and diarrhea were enrolled and, using the glucose breath test, a prevalence rate of SIBO of 38.5% was found. Moreover, in this study a 75 g dose of glucose was administered and such a high carbohydrate load could be responsible for a significant osmotic effect causing a high number of false positive results, especially in a subset of diarrhea patients.

Finally, the pathophysiological role of SIBO in IBS was disproved in a recent paper [30], which analyzed the prevalence of SIBO in IBS patients, by adopting the test considered the gold standard for its diagnosis, *i.e.*, the culture of jejunal aspirates. This paper showed the same prevalence (4%) of positive tests in both IBS and controls. However, by using a cut-off positivity value of 10^3^ CFU/mL of jejunal aspirate, instead of 10^5^, IBS patients showed a significantly higher prevalence of positivity results. Two major criticisms may be raised against these results: first, jejunal aspirate was performed through the central channel of a manometric catheter, thus without adopting methods that could exclude a possible contamination by oro-pharyngeal flora, that may be responsible for a descending contamination; second, no study is available showing that the presence of 10^3^ CFU/mL in jejunal aspirate causes an alteration of small bowel intraluminal micro-environment, responsible for malabsorption and symptom onset. In this light, the absence of clinical symptoms in control subjects harbouring 10^5^ CFU/mL of jejunal aspirate raises many doubts on the ability of 10^3^ CFU/mL of jejunal aspirate to cause symptom onset. There is no doubt that functional bowel disorders and SIBO have a clinical overlap, but this is not sufficient to hypothesize a pathophysiological overlap.

## 5. The Rationale for the Use of Antibiotics in IBS

IBS is characterized by a multifactorial pathophysiology [31]. It is, therefore, improbable that the same therapeutic approach will show the same efficacy in all patients. Accordingly, the right selection of patients to start different pharmacological approaches is crucial: the wrong selection is certainly the cause of erroneous therapeutic approaches and poor outcomes. Moreover, the inappropriate use of a drug can lead to a generic judgment of inefficacy of the drug, which could subsequently lead to it not being used, even in patients who are theoretically able to respond. 

In our opinion, at least one subgroup of IBS patients will benefit from antibiotic treatment and, if accurately selected, the severity of their symptoms will improve. According to Levitt’s results [17], in IBS patients suffering from moderate-severe flatulence the pathophysiology of this symptom must be correlated to an excessive intestinal gas production. Thus, these patients will show an improvement of flatulence severity after antibiotic treatment. We have previously shown that the administration of rifaximin in functional patients improves the severity of flatulence and this improvement is strictly correlated to the reduction of intestinal gas production [12]. Hence, the presence of moderate-severe flatulence is positively affected by the administration of antibiotics, associated with a reduction of the intake of fermentable fibers with diet. 

The same direct correlation between gas production and symptom is not always true for bloating. This symptom may be caused by many different mechanisms [32] and the modification of intestinal gas production may not always be appropriate. Generally, bloating associated with flatulence is prevalently linked to the same mechanism and, therefore, it would be expected to improve with antibiotic therapy. If flatulence is not excessive, the treatment for bloating should probably be based on other pathophysiological mechanisms.

## 6. Conclusions

The treatment of IBS is a difficult task for clinicians. A rigid protocol, with one single therapeutic regimen for all patients is certainly questionable. Rather, a more flexible approach, tailored to the patient represents a more accurate approach. If gas-related symptoms are present and contribute to reducing quality of life, antibiotics may be administered in this selected group.
